# Quantum dots: synthesis, bioapplications, and toxicity

**DOI:** 10.1186/1556-276X-7-480

**Published:** 2012-08-28

**Authors:** Alireza Valizadeh, Haleh Mikaeili, Mohammad Samiei, Samad Mussa Farkhani, Nosratalah Zarghami, Mohammad kouhi, Abolfazl Akbarzadeh, Soodabeh Davaran

**Affiliations:** 1 Department of Medical Nanotechnology, Faculty of Advanced Medical Science, Tabriz University of Medical Sciences, Tabriz, 51664, Iran; 2 Department of Physics, Tabriz Branch, Islamic Azad University, Tabriz, Iran; 3 Faculty of Dentistry, Tabriz University of Medical Sciences, Tabriz, 51664, Iran; 4 Tuberculosis and Lung Disease Research Center of Tabriz University of Medical Sciences, Tabriz, 51656-65811, Iran

**Keywords:** QD delivery systems, Toxicity, Emission spectra, Luminescence characteristics

## Abstract

This review introduces quantum dots (QDs) and explores their properties, synthesis, applications, delivery systems in biology, and their toxicity. QDs are one of the first nanotechnologies to be integrated with the biological sciences and are widely anticipated to eventually find application in a number of commercial consumer and clinical products. They exhibit unique luminescence characteristics and electronic properties such as wide and continuous absorption spectra, narrow emission spectra, and high light stability. The application of QDs, as a new technology for biosystems, has been typically studied on mammalian cells. Due to the small structures of QDs, some physical properties such as optical and electron transport characteristics are quite different from those of the bulk materials.

## Review

### Introduction

In the past years, a new class of fluorescent particles emerged as a good candidate for single molecule and single particle tracking (SPT) in living cells and organisms, the semiconductor quantum dots [1]. Quantum dots (QDs), often described as ‘artificial atoms,’ exhibit discrete energy levels, and their bandgap can be precisely modulated by varying the size [2]. QDs are nanometer-scale semiconductor crystals composed of groups II to VI or III to V elements and are defined as particles with physical dimensions smaller than the exciton Bohr radius [3]. QDs exhibit unique luminescence characteristics and electronic properties such as wide and continuous absorption spectra, narrow emission spectra, and high light stability [4]. They absorb white light and then re-emit a specific color a few nanoseconds later depending on the bandgap of the material [5-7]. QDs are one of the first nanotechnologies to be integrated with the biological sciences [4,8] and are widely anticipated to eventually find application in a number of commercial consumer and clinical products [9]. For example, CdSe/ZnS quantum dots are presently the most common commercially available product as secondary antibody conjugates that are composed of a core of cadmium selenide ranging from about 10 to 50 atoms in diameter and about 100 to 100,000 atoms in total [10]. QD range is typically between 2 and 10 nm in diameter. QDs consist of a semiconductor core, overcoated by a shell (e.g., ZnS) to improve optical properties, and a cap enabling improved solubility in aqueous buffers [11]. The application of QDs, as a new technology for biosystems, has been mostly studied on mammalian cells. There is an increasing tendency to apply QDs as markers in plant science [12-16]. The application of QDs as markers of the cells or their cell walls for plant bioimaging would be advantageous because of their small size, brightness, independence of emission on the excitation wavelength, and stability under relatively harsh environments. They also have excellent photostability [17] and overcome the limitations associated with photobleaching. Due to the small structures of QDs, some physical properties such as optical and electron transport characteristics are quite different from those of the bulk materials [18]. The study of the impurity states in these low dimensional structures is an important aspect to which many theoretical and experimental works based [16,19-21]. This review introduces QDs and explores their properties, synthesis, applications, delivery systems in biology, and their toxicity.

### Synthesis

Several routes have been used to synthesize QDs [22] but, generally, techniques for QD synthesis used top-down processing methods and bottom-up approach. Top-down processing methods include molecular beam epitaxy (MBE), ion implantation, e-beam lithography, and X-ray lithography. Using the alternative bottom-up approach, colloidal QDs are prepared by self-assemblyin the solution following a chemical reduction [23-26].

In the approaches of top-down, for making the QDs, a bulk semiconductor is thinned. For the achieve QDs of diameter approximately 30 nm, electron beam lithography, reactive-ion etching, and/or wet chemical etching are commonly used. For systematic experiments on quantum confinement effect, controlled shapes and sizes are achievable with the desired packing geometries. Alternatively, focused ion or laser beams have also been used to fabricate arrays of zero-dimension dots. Incorporation of impurities into the QDs and structural imperfections by patterning are major disadvantages with these processes [22].

A number of different self-assembly techniques (bottom-up) have been used to synthesize the QDs, and they may be broadly subdivided into wet-chemical and vapor-phase methods [22]: (a) wet-chemical methods mainly follow the conventional precipitation methods with careful control of parameters for a single solution or mixture of solutions. The precipitation process invariably involves both nucleation and limited growth of nanoparticles. Nucleation may be categorized as homogeneous, heterogeneous, or secondary nucleation [27]. Homogeneous nucleation occurs when solute atoms or molecules combine and reach a critical size without the assistance of a pre-existing solid interface. Wet-chemical methods are generally microemulsion, sol–gel [28-30], competitive reaction chemistry, hot-solution decomposition [31-33], sonic waves or microwaves [34], and electrochemistry. (b) Vapor-phase methods for producing QDs begin with processes in which layers are grown in an atom-by-atom process. Consequently, self-assembly of QDs occurs on a substrate without any patterning [35-38]. Self-assembly of nanostructures in material grown by MBE, sputtering, liquid metal ion sources, or aggregation of gaseous monomers are generally categorized under vapor-phase methods [22]. MBE has been mainly used to self-assemble QDs from III-V semiconductors and II-VI semiconductors using the large lattice mismatch, e.g., InAs on GaAs has a 7% mismatch and leads to SK growth [35].

### Applications

In this review, we evaluate few experiments that show the high potential of QDs in biological application, including tracking different macromolecules in the cell, tracking various cells in the tissue, labeling organelles and cells, clinical applications, and other applications [39-43].

#### QDs for labeling cells

Because QDs have constant and unique optical properties, they are the best candidate for cell labeling, as compared with organic dyes.

*Use in plant bioimaging* There is an increasing application of QD as markers for the cells or cell walls (CWs) in plant science. A first target location for external agents in a plant cell is the CW [44]. Djikanović et al. demonstrated that CdSe QDs bind typically to cellulose and lignin in the cell wall of *Picea omorika* branch. Respectively, binding to lignin and cellulose are achieved by interaction with the chains of C = C and C-C alternating bonds and interaction with the OH groups [44]. Data showed that QDs are suitable for homogenous marking of the whole cell wall. This is a consequence of the structural arrangement of the cell wall polymers in the whole cell wall network as well as the extremely small size of the QDs. These characteristics enable a feasible penetration of the nanoparticles inside the polymer structures in the CW composite [44].

**Figure 1:**
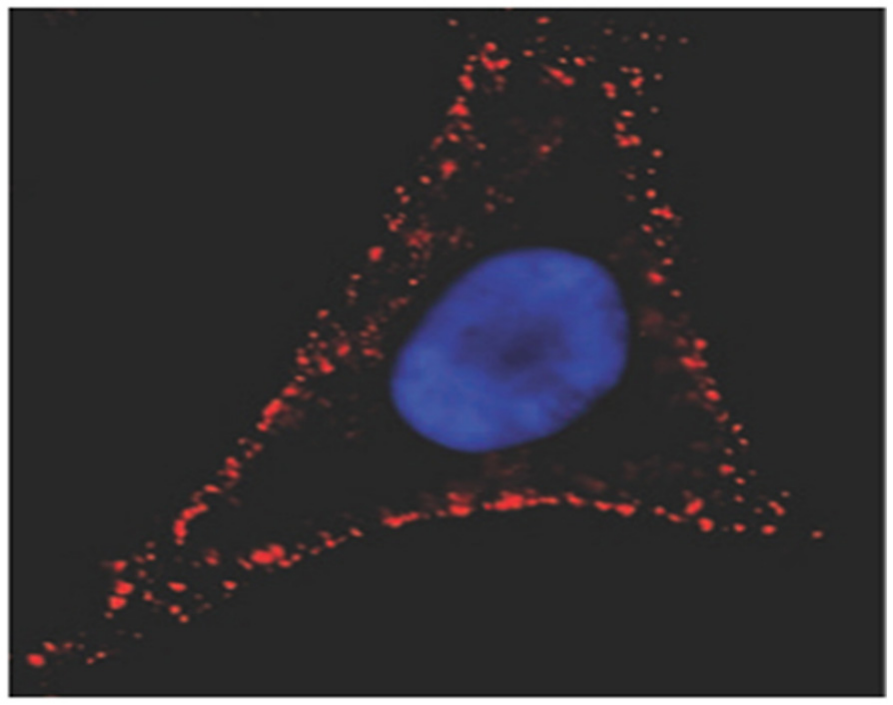
**Live HeLa cells growing on a glass coverslip.** Labeled with QD-avidin for GM1 (in red) and Hoechst 3342 for nuclear staining (in blue) [46].

*Use in animal bioimaging* Goldman et al. used biotinylated CTxB in conjunction with QD-avidin conjugates [45] for labeling of the live HeLa cells which Figure 1 shows an image of the lateral membrane staining for GM1 ganglioside using QDs (in red) and nuclear staining using Hoechst (in blue). Punctuate labeling of the cell surface by QD bioconjugate is typical for molecules such as GM1 that is present in membrane rafts [46].

**Figure 2:**
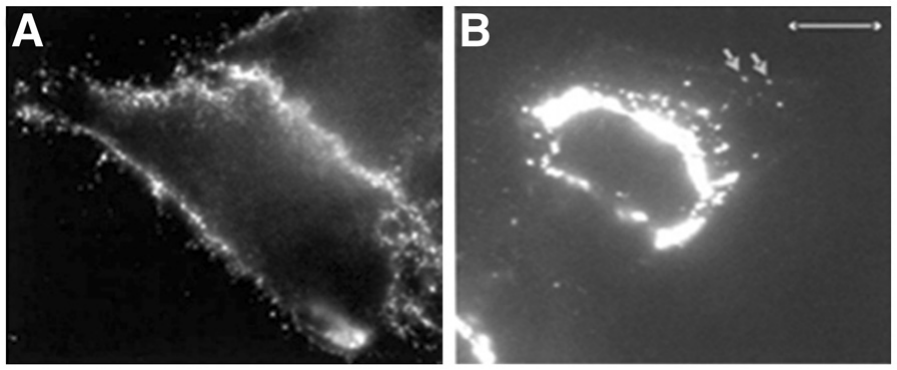
**HeLa cells labeled with the avidin-conjugated yellow-emitting QDs.**[47]. (**A**) Image of cells immediately after the unbound QDs were removed in which labeling is restricted to the cell surface. (**B**) Image of a cell that was allowed to grow for 2 h after washing out of unbound QDs.

In another study, they labeled live HeLa cells which were biotinylated using sulfo-NHS-SS biotinylating reagent and then incubated with the avidin-conjugated yellow-emitting QDs. It is shown in Figure 2[47].

For long-term live cell imaging, Hasegawa et al. used the CHPNH_2_-QD complexes which were uniformly internalized into the cells without being aggregated. Therefore, CHPNH_2_ nanogel has high potential for use in long-term live cell imaging. The interaction of QDs with cells was successfully controlled by the amino group content of the CHPNH_2_ nanogel [48].

**Figure 3:**
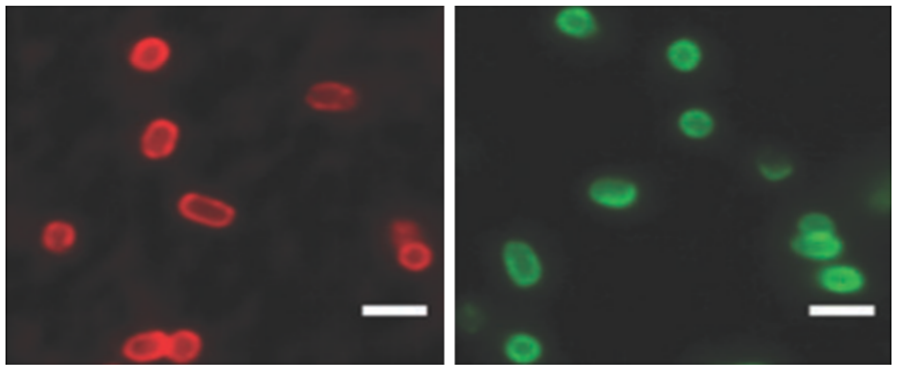
**Imaging of rough *Escherichia coli* JM83 cells.** Left imaging is red QD, and right imaging is green QD. Scale bar is 2 μM [49].

*Use in prokaryote bioimaging* Sensitive and selective staining of bacterial mutants using QD labels was demonstrated by Smith's group. This principle of detection is based on selective targeting affinity of Zn(II)-dipicolylamine coordination complex to phospholipids on the bacterial cell surface of specific strain as shown in Figure 3[49,50].

In another study, authors demonstrated the use of magnetic beads coated with anti-*E.coli O157* antibodies and streptavidin-coated QDs for measuring the bacterial cell concentration [51]. Yang and Li, using QDs with different emission wavelengths (525 nm and 705 nm), reported the simultaneous detection of *E. coli O157:H7* and *Salmonella typhimurium*[52].

#### Tracking different particles

With the application of new imaging methods and the use of brighter and more stable probes, such as QDs, single particle tracking has the potential to enter into a new era of high resolution and long timescale imaging [53-55]. SPT techniques allow scientists to follow single molecules in real time and visualize the actual molecular dynamics in their habitant environment.

*For extracellular study* Because QDs do not require intracellular delivery through the impermeable plasma membrane, membrane receptors or membrane-associated proteins are intuitive targets for QD imaging [53]. Howarth et al. demonstrated a method to track endogenous cell-surface proteins without cross-linking by purifying monovalent antibody-QD conjugates. They approach to make monovalent tight-binding QDs, using mSA, which could be applied to other nanoparticles that show sufficient electrophoretic mobility. They applied sQD-mSA1 to study the mobility of a mutant of low-density lipoprotein (LDL) receptor with a truncated cytosolic tail, originally found from an individual with familial hypercholesterolemia. This mutant phenotype has been extensively investigated by following LDL, but Howarth and co-workers analyzed the behavior of the receptor itself (supplementary methods). They imaged single monovalent sQDs bound to the biotinylated AP-LDL receptor, as indicated by QD fluorescence intensity and blinking. The mobility of mutant receptors labeled with sQD-mSA1 was significantly greater than that of labeled wild-type LDL receptor (*P* = 1.6 × 10^−14^) [56].

In similar studies, recently, QDs used to target membrane proteins and investigate the mobility and entry-exit kinetics in several systems: (1) various transmembrane proteins, for example, integrins [57], channels [58], and aquaporines [59]; (2) receptors GABA [60], glycine [61], interferon [62], and HER [63,64]; and (3) neurological synapse [65,66].

**Figure 4:**
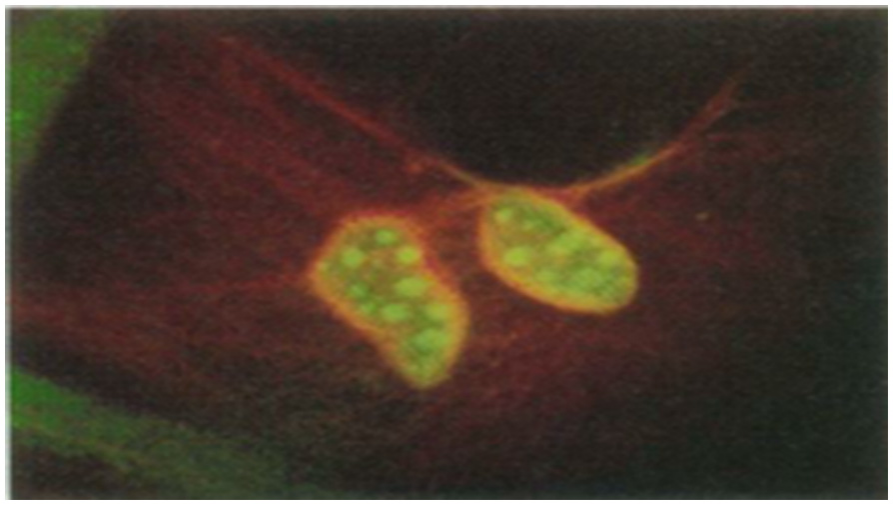
Image was obtained with 363-nm excitation and × 40 oil 1.3 numerical aperture objective.

*For intracellular study* In one of the study, the advantages of the broad, continuous excitation spectrum were demonstrated in a dual-emission, single-excitation labeling experiment on mouse fibroblasts. These nanocrystal probes are, thus, complementary and, in some cases, may be superior to existing fluorophores [4]. Nonspecific labeling of the nucleus by both the red and the green probes resulted in a yellow color. The red actin filaments were specifically stained. Also, the green probes penetrate into the nucleus. Both are shown in Figure 4[4].

This is shown as green color for nucleus and red color for actin filaments. Nonspecific labeling of the nucleus by both the red and the green probes resulted in a yellow color [4].

**Figure 5:**
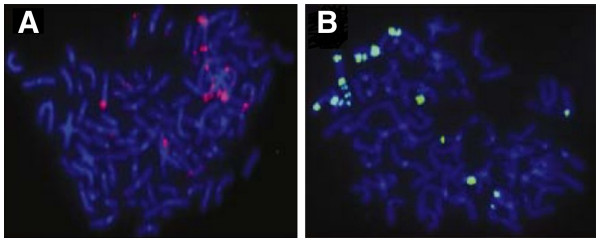
**Qualitative FISH detection of HER2 gene-amplified SK-BR-3 breast cancer cells.** With (**A**) streptavidin-conjugated Qdot605 and (**B**) FITC, respectively [67].

Superior stability of QD fluorophores gives the possibility to improve quantitation of FISH analysis of human chromosomal changes. Xiao and Barker have investigated coated (CdSe)ZnS QDs as fluorescence labels for FISH of biotinylated DNA to human lymphocyte metaphase chromosomes under conditions that approximate those commonly found in clinical cytogenetics laboratories [67]. They have also demonstrated the application of QDs to FISH detection of the clinically relevant HER2 locus in breast cancer cells (Figure 5).

Pierobon et al. [68] and Nelson et al. [69] tagged myosin V molecules with QDS toestablish a link between *in vitro* and in-cell measurements of myosin V motors. Then, the complex myosin V/QD (MyoV::QD), using the pinocytic influx, was introduced into the cells.

Yoo et al. [70] and Courty et al. [71] characterized the dynamics of other major actors of intracellular transport: the kinesin-1, the actin filaments, and the microtubules [65].

#### Imaging in situ

**Figure 6:**
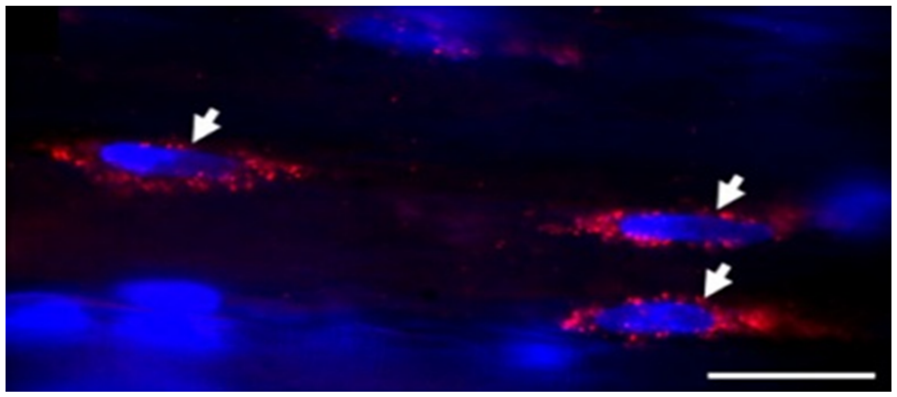
**Double fluorescence staining to visualize the localization of M-cadherin (in red) and nuclei (in blue).** Arrows indicate that M-cadherin-positive satellite cells were located within the intact soleus muscle *in situ*[72].

*Imaging of the satellite cells in rat intact and injured soleus muscles using quantum dots* The employment of satellite cells, which are located between the basement membrane and the plasma membrane in myofibers, is required for myofiber repair after muscle injury or disease. Using QDs conjugated to anti-M-cadherin antibody, Ishido and Kasuga attempted the visualization of satellite cells in both intact and injured skeletal muscle of rat *in situ*. They demonstrated *in situ* real-time imaging of satellite cells localized within the skeletal muscle (Figure 6) [72].

*Imaging morphogenesis in Xenopus with quantum dot nanocrystals* Stylianou and Skourides are the first to report the use of near-infrared QDs to image mesoderm migration *in vivo* with single cell resolution and provide quantitative *in vivo* data regarding migration rates [73].

**Figure 7:**
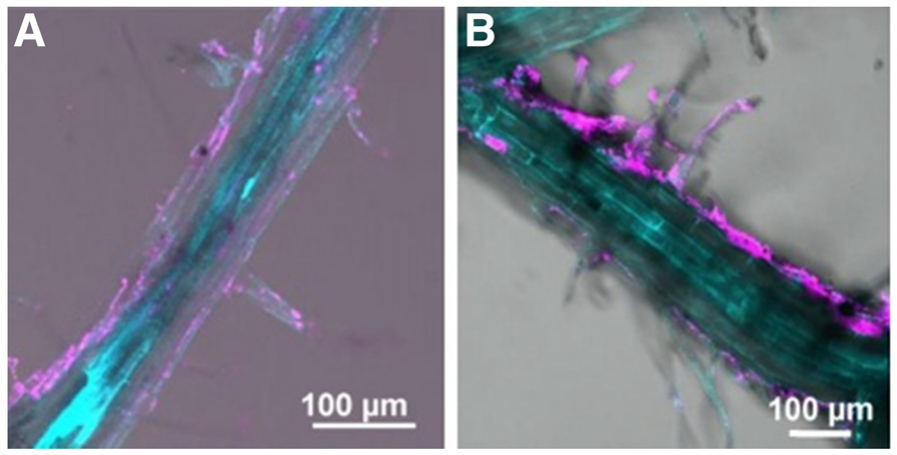
**Imaging of roots from plants exposed to QD suspension in HS + HA**. For (**A**) 1 and (**B**) 7 days [74].HS, Hoagland's solution; HA, one of the important groups of organic acids.

Navarro et al. experiments revealed that *Arabidopsis* exposed to QDs that are dispersed in Hoagland's solution for 1 to 7 days did not internalize intact QDs. Fluorescence microscopy showed strong evidence that the QDs were generally on the outside surfaces of the roots (Figure 7). The amount of QDs adsorbed is dependent on the stability of the QDs in suspension [74].

#### Using QDs in clinical applications

The development of multifunctional nanomaterials combining diagnostic and therapeutic purpose has recently attracted intensive interests [75-81]. In this paper, we have reviewed the clinical applications of QDs in the three categories that include: (1) biomarker detection in various cancers, (2) imaging and sensing of infectious diseases, and (3) other clinical therapeutic applications.

*Biomarker detection in various cancers using QDs* The detection of cancer biomarkers is important for diagnosis, disease stage forecasting, and clinical management [82]. QDs with intense and stable fluorescent properties could enable the detection of tens to hundreds of cancer biomarkers in blood assays, on cancer tissue biopsies, or as contrast agents for medical imaging. Clinical outcome of cancer diagnosis is highly dependent on the stage at which the malignancy is detected, and therefore, early screening has become extremely important in any type of cancer [83].

 1. Multicolor and multiplexing potentialities of QDs are used for the detection of four protein biomarkers CD15, CD30, CD45, and Pax5 of Hodgkin's lymphoma from lymphoma tissues. Simultaneous visualization using multiplexed QD staining was advantageous for the selective identification of rare Hodgkin (Reed-Sternberg) cells, a primary diagnostic target for Hodgkin's disease, which was not achievable using traditional immunohistochemistry assays [84,85].

 2. Yu et al. reported the use of GSH-TGA-QDs-ND-1 probes to label colorectal cancer cells CCL187. They prepared QDs, which were conjugated with monoclonal antibody ND-1 for specific reaction with antigen LEA [86].

 3. In the United States, pancreatic cancer is the fourth leading cause of cancer death (about 18,770 men and 18,030 women (36,800 people) in 2010) [87]. Using semiconductor QD-antibody conjugates, Lee et al. demonstrated quantitative profiling of biomarkers for pancreatic cancer at the single-cell level. Their results show the possibility of this method for staging and forecasting, such as prostate stem cell antigen claudin-4, and mesothelin, which are expressed in different stages of progression of pancreatic cancer [82]. Anyway, realizing quantitative profiling requires stable quantum yield, monodisperse QD-Ab conjugates, and well-defined surface chemistry [88].

There are evidences showing the application of QDs in micro- and nanoarrays for the detection of cancer biomarkers [83].

*Imaging and sensing of infectious diseases by QDs* QDs have become one of the most hopeful and interesting materials for diagnostic applications of bioimaging, labeling, and sensing for infectious diseases such as respiratory syncytial virus (RSV)that isone of the families of Paramyxoviridae [50]*. *In Table 1, some of the infectious diseases and QDs used to distinguish them are shown.

**Table 1:** Some of the infectious diseases and QDs used to distinguish them

**Authors**	**Type of infectious diseases**	** *In vitro* / *in vivo* **	**Type of modified QDs**
Tripp et al. [89]	RSV	*In vitro*/*in vivo*	Antibody anti-F protein conjugated to QDs(CdTe)
Agrawal et al. [90]	Individual molecules of genes, proteins, and virus particles	*In vivo*	QD-antibody color-coded NP probes and two-color co-localization imaging
Bentzen et al. [91]	RSV	*In vivo*	Streptavidin-coated QDs conjugated to antibody anti-F and antibody anti-G
Dwarakanath et al. [92]	*S. typhimurium*	*In vivo*	Antibody-QD and DNA aptamer-QD
	*E. coli*		
	*B. subtilis* spores		
Goldman et al. [93]	Choleratoxin, ricin, shinga-like toxin1 and staphylococcal enterotoxin B	*In vitro*	Antibody-QD (CdSe/Zns)
Zhao et al. [94]	Food-borne pathogenic *E. coli* O157:H7, *S. typhimurium* and *S. flexneri*		QDanti-*S. flexneri* antibody,anti-*E. coli* antibody, anti-*S. typhimurium* antibody
Hahn et al. [95]	Single cells of *E.coli* O157:H7		Streptavidin-coated QDs conjugated to antibody
Mukhopadhyay et al. [96]	Detect *E. coli* at levels as low as 10^4^ bacteria/ml of sample		Mannose-conjugated QDs
Edgar et al. [97]	*Mycobacterium*	*In vivo*	Streptavidin-coated QDs conjugated to phage
	*B. anthracis*		
Zhu et al. [98]	*C. parvum*		QD-conjugated antibodies
	*G. lamblia*		
Klostranec et al. [99]	Biomarkers of the most globally prevalent blood-borne infectious diseases (i.e., hepatitis B, hepatitis C, and HIV) with low sample volume		QD-antibody
Gazouli et al. [100]	*Mycobacterium* genus	*In vivo*	Specific DNA sequences combining QDs with magnetic beads [101,102]
Hahn et al. [95]	Individual pathogenic *E. coli O157:H7* in phosphate buffer saline solution		Streptavidin-coated Qdots labeled by antibody selectively targeted pathogenic *E. coli* O157:H7
Su and Li [51]	*E. coli O157*	*In vivo*	Streptavidin-coated QDs conjugated to anti-*E. coli* O157 antibody
Yang and Li [52]	*E. coli* O157∶H7	*In vivo*	QDs with different sizes conjugated to anti-*E. coli* O157 and anti-*Salmonella* antibodies
	*S. typhimurium*		
			The bead-cell complexes reacted with QD-antibody conjugates to form bead-cell-QD complexes

Other clinical therapeutic applications.

 1. QDs for assessing axon growth

**Figure 8:**
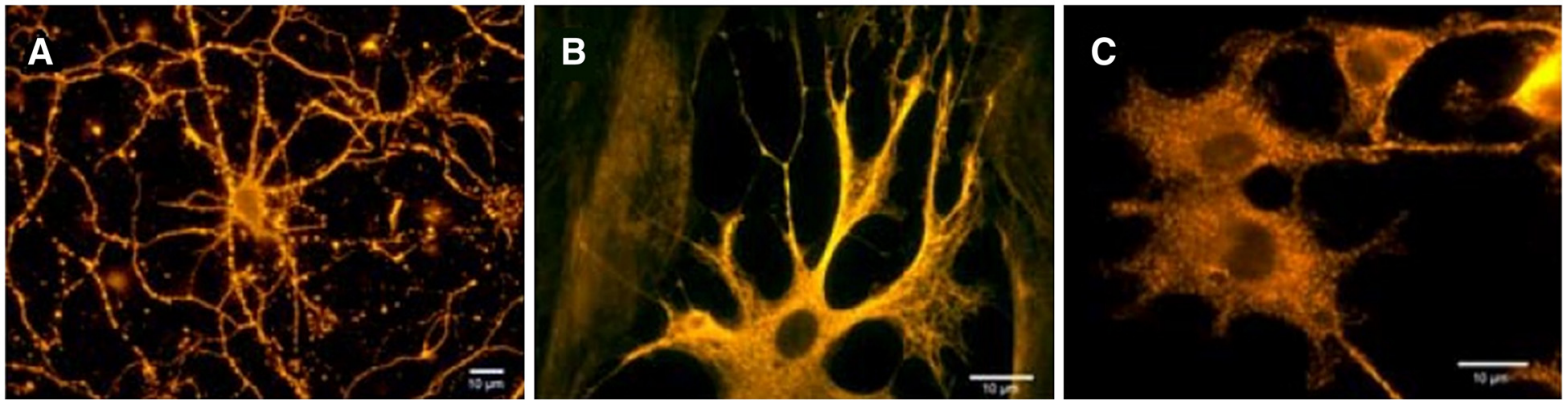
**Using QD conjugated with antibody for labeling of neurons and glia.** (**A**) Labeled β-tubulin in primary cortical neurons. (**B**) Labeled glial fibrillary acidic protein in primary cortical astrocytes. (**C**) Labeled for β-tubulin in PC12 cells [106].

A major health problem with injuries to the spinal cord and brain is traumatic central nervous system injury reporting of approximately 265,000 and 1.5 million new injuries each year [103-105]. QDs represent a new device of significant potential in neuroscience research, and they are useful for experiments that are limited by the restricted anatomy of neuronal and glial interactions [106]. One of the problems in treatment is estimating its effectiveness. They allow the ability to visualize and track dynamic molecular processes over long times (Figure 8) [106]. Application of surface-engineered QDs is an area of nanotechnology probing the details of cellular and molecular processes in neuronal cells [4,107-109]. QD bioconjugates based on surface chemistry can be broadly classified as follows: (1) QDs' surface modified by bioactive molecules and (2) QD-polymer nanocomposites [103]. This advance might be significantly important to assess axon growth pending the regeneration process [103]. Previous investigations were demonstrated in Table 2.

**Table 2:** Applications of QDs in labeling neurons and glia cells

**Authors**	**Type of QD used**	**Application of QD**
Dahan et al. [61]	QD-GlyR	Target neurons to investigate a specific neurophysiological process(QDs to track individual glycine receptors and analyze their lateral dynamics in the neuronal membrane)
Pathak et al. [106]	Antibody-conjugated quantum dots	Performed the specific labeling of neurons and glia cells
Vu et al. [110]	Tagged nerve growth factor (βNGF) to QDs	Investigate the QD nanostructure's potential to assess the neurite outgrowth
Sundara Rajan et al. [111]	QD-anti-TrkA-TrkA receptor with transport by GFP	Immobilized QDs were conjugated with NGF, activate Trk receptors, and initiate neuronal differentiation in PC12 cells.
Howarth et al. [112]	Tagged cell surface proteins with a specific peptide (acceptor protein) that can be directly biotinylated as a target for streptavidin-conjugated quantum dots	Specifically label and track AMPA receptors on cultured hippocampal neurons
Prasad et al. [113]	Thioglycolic acid (TGA)-stabilized CdTe QDs	Performed imaging of PC12 cells

 2. QD used as a probe in an anti-malarial drug-screening assay

Malaria is a major global health problem, threatening over 300 million people and causing nearly one million deaths annually [114,115]. Tokumasu et al. used QD-Ab to demonstrate the distinct pattern of distribution of protein and to observe erythrocyte membrane deformation occurring duringthe invasion of erythrocytes by *Plasmodium falciparum*[116]. Ku et al. showed a simple and efficient method to label *P. falciparum*-infected RBC using a QD-based probe and its applicability as an efficient probe for anti-malarial drug screening [115].

#### Other applications

*QDs as pH probes for the study of enzyme reaction kinetics*[117] Lately, worth advancement has been achieved in water-soluble QDs as ionic probe. Jin et al. reported the use of modified CdSe QDs for the sensitive determination of cyanide ions [C ≡ N]^−^[117,118]. Xie et al. reported the determination of Cu^2+^ by using CdSe/ZnS QDs modified with bovine serum albumin [119]. QDs also have been reported to be sensitive to pH [120-125]. The sensitivity of QDs' photoluminescence to pH, improve stability, and a monitoring range for the determination of proton concentration, which is maybe due to a function of surface modifications and effects on exciton trap sites, leads to applications utilizing QDs as pH probes [126]. Water-soluble QDs, ZnS, modified with mercaptoacetic acid (MAA) were sensitive to environmental factors and found to be a satisfactory pH probes that could have potential applications in chemical and biochemical sensing. Using the modified QD surface, they were applied as pH probes in monitoring the hydrolysis of glycidyl butyrate which is catalyzed by porcine pancreatic lipase (PPL) [117].

*QDs use for protein micro- and nanoarrays to the detection of cancer biomarkers* Protein microarrays are useful device as highthroughput screening tools in proteomics [127-129], for biosensing purpose [130], new drug discovery [131], and enabling a quick parallel screening method for the detection of protein-protein interactions in case of large protein populations. There are various reports in which QDs have been used in microarray fabrication such as sandwich-based immunoassay type, RP protein microarray type, etc. [132-135]. Here, IgG detection was done on a glass chip using a QD-labeled secondary Abs as sandwich assay approach. In RP protein microarrays, Geho et al. used pegylated QDs conjugated with streptavidin as detection elements. In another study, Zajac et al. investigated the ability of the platform to detect different cytokines TNF-α, IL-8, IL-6, MIP-1β, IL-13, and IL-1β using two different models of quantum dot probes. Their results demonstrated high sensitivity of the investigated detection system with less than picomolar concentration [136]. Kerman et al. reported the use of QDs for detection cell lysates spiked with DNA-PK proteins with the help of mAb, in an RP protein microarray format. Kerman et al. make immunosensor based on QD for the detection of prostate specific antigen (PSA) in a sandwich assay approach for chip fabrication [134]. Gokarna et al. used pegylated QD-conjugated PSA Abs to demonstrate the fabrication of a cancer protein biochip for the detection of PSA, which is a biomarker for prostate cancer. The QD nonspecificity can show to be quite detrimental to some extent in case of multiplexed assay systems where multiple proteins are to be detected simultaneously [83].

*QD delivery* Due to the unique properties of QDs, they are best tools for intracellular studies such as visualizing the cellular structure, studying the dynamic cellular processes, and tracking single molecules in the cell [137,138]. To achieve this goal, translocation of functionalized QDs into the cell for labeling organelles and tracking single molecules is important. QDs have hydrophobic surface and have a little toxicity, therefore cannot be applied *in vivo* unless their surface is modified. Thus, by surface modification, their hydrophilicity will increase but their toxicity will decrease.

**Figure 9:**
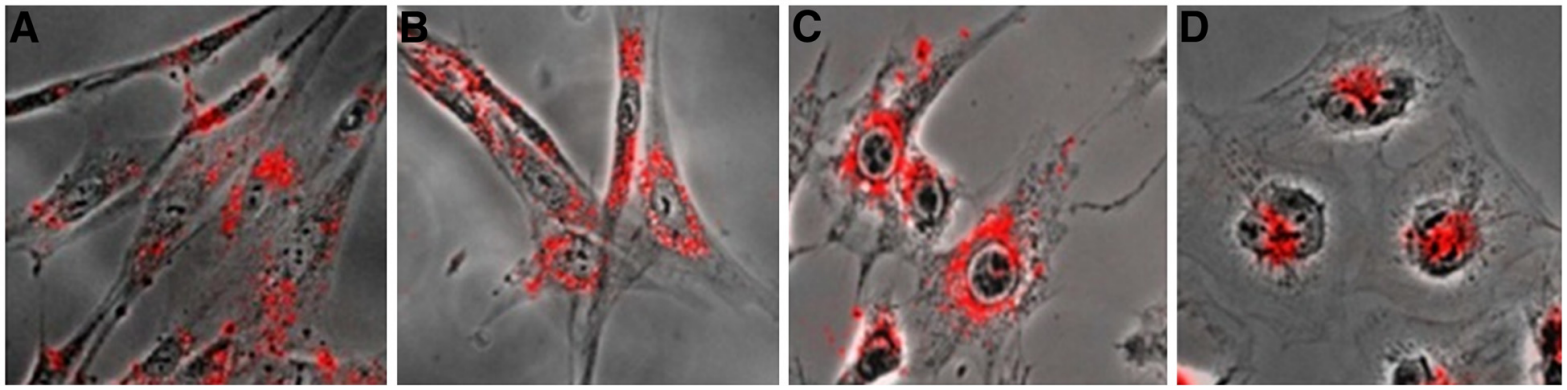
**Confocal laser scanning fluorescence microscopyimages of cells labeled with CHPNH**_**2**_**(15)-QD nanoparticle.** (**A**) TIG-3 cells, (**B**) MRC-5 cells, (**C**) MCF-7 cells, and (**D**) YKG-1 cells [48].

Hasegawa et al. used nanogel-QD hybrid nanoparticles for live cell imaging [48]. They also confirmed the cellular uptake of CHPNH_2_(15)-QD nanoparticles using other normal cells (TIG-3 and MRC-5) and cancer cells (T24, Saos-2, T98G, A549, MCF-7, and YKG-1) (Figure 9) [48].

In recent years, functional peptides that transmit biomaterials into cells have been developed in biomaterial research. Because of lysosomal trapping, QD delivery into cells with conjugated cell-penetrating peptides by the endocytic pathway was challenging in biomedical applications [139]. In another study, engineered peptides for producing QDs tagging protein ligands and biosensors to their surfaces, by appropriate cysteines or histidines, have served as ligands [140]. Encapsulation of QDs in viral capsids provides a new tool which allows the design of intracellular microscopic probes and vectors [141]. More samples of QD delivery systems are shown in Table 3.

**Table 3:** QD delivery systems

**Authors**	**Delivery system of QD**	**Use**
Jia et al. [142]	Multiwalled carbon nanotube (MWNT) delivery system	MWNTs are containing antisense oligodeoxynucleotides and CdTe QDs via electrostatically layer-by-layer assembling.
Chen et al. [143], Xue et al. [144], Delehanty et al. [145], Ruan et al. [146], Wei et al.[147]	Tat peptide-mediated delivery system	QDs conjugated to the cell-penetrating peptide derived from the human immunodeficiency virus-1 transactivator protein
Lagerholm et al. [148]	Peptide delivery system	Nine residue biotinylated l-arginine peptide is used to enhance delivery of streptavidin-conjugated QDs into mammalian cells.
Bagalkot et al. [149]	A10 RNA aptamer	Functionalizes the surface of QD with the A10 RNA aptamer, which recognizes the extracellular domain of the prostate specific membrane antigen
Bakalova et al. [150]	Silica-shelled quantum dots	Based on silica-shelled single QD micelles with incorporated paramagnetic substances [tris(2,2,6,6-tetramethyl-3,5-heptanedionate)/gadolinium] into the micelle and/or silica coat
Yum et al. [151]	Nanoscale mechanochemical method	Using a membrane-penetrating nanoneedle
Yuan et al. [152]	Chitosan (N-acetylglucosamine) tumor-targeted drug delivery	QDs encapsulated with chitosan
Hasegawa et al. [48]	Nanogel-QD hybrid	Nanogels of CHPNH 2 with 15 amino groups per 100 glucose units and QDs that were conjugated with protein A molecules were mixed.
Dixit et al. [141]	Viral vectors	QDs encapsulation in viral capsids
Zhang and Liu [153]	Nonviral vectors	Cappingthe surface of ZnO QD with poly(2-(dimethylamino) ethyl methacrylate)
Jablonski et al. [154]	Cationic peptide and a hydrophobic counterion	Quantum dots have been delivered to the cytosol of living cells using a combination of a cationic peptide, polyarginine, and a hydrophobic counterion, pyrenebutyrate.
Qi and Gau [155]	QD-amphipol nanocomplex	Advantages include cytoplasm delivery and endosome escape.
Gao et al. [109]	Polymeric delivery system	The structural design involves encapsulating QDs with an ABC triblock copolymer and linking this amphiphilic polymer.
Duan and Nie [77]	Polymeric delivery system	QDs were encapsulated by PEI-g-PEG.

### Toxicity of QDs

There are different opinions about the toxicity of QDs; therefore, we investigated their toxicity in amoeba as primary eukaryotes, in plant, and in animal.

#### In amoeba

It has been determined that QD labeling had no detectable effect on cell growth and had no deleterious effects on cellular signaling and motility during development of the *Dictyostelium discoideum* cells [47].

#### In plant

The ratio of reduced glutathione levels (GSH) relative to the oxidized glutathione (GSSG) in plants suggests that QDs caused oxidative stress on the plant at this condition [74].

#### In animal

Yan et al. investigated the potential vascular endothelial toxicity of mercaptosuccinic acid (2-sulfanylbutanedioic acid)-capped QDs *in vitro*. Their results suggested that QDs could not only impair mitochondria but also exert endothelial toxicity through activation of mitochondrial death pathway and induction of endothelial apoptosis [156].

More recently, Chen et al. have studied the cytotoxicity of CdTe/CdS (core-shell) structured and also CdTe/CdS/ZnS (core-shell-shell) structured aqueous synthesized QDs, and their results suggest that the cytotoxicity of CdTe QDs not only comes from the release of Cd^2+^ ions but also intracellular distribution of QDs in cells and the associated nanoscale effects [157]. Table 4 demonstrated more results for toxicity of QDs [158-162].

**Table 4:** **More details for toxicity of QDs (modified from**[163]**)**

**QD**	**Model**	**Administration**	**QD concentration**	**Exposure duration**	**Toxicity**	**Study**
CdSe/ZnS-SSA	EL-4 cells	1 × 10^6^ cells/well	0.1 to 0.4 mg/mL	0 to 24 h	Cytotoxic: 0.1 mg/mL altered cell growth; most cells nonviable at 0.4 mg/mL	Hoshino et al. 2004a
CdSe/ZnS-SSA	EL-4 cells	200-μL cell suspension injected (iv) into the mice	0.1 mg/mL QDs per 5 × 10^7^ cells	2 h to 7 days	No toxicity in mice *in vivo*	Hoshino et al. 2004a (*in vivo*)
CdSe/ZnS conjugates: NH_2_, OH, OH/COOH, H_2_/OH, MUA, COOH	WTK1 cells	5 × 10^4^ cells/mL	1 to 2 μM	12 h	2-μM QD-COOH-induced DNA damage at 2 h	Hoshino et al. 2004b
CdSe/ZnS-MUA	Vero, HeLa, and primary human hepatocytes	100-μL QDs/3 × 10^4^ cells	0 to 0.4 mg/mL	24 h	Cytotoxic: 0.2 mg/mL, Vero; 0.1 mg/mL, HeLa; 0.1 mg/mL, hepatocytes	Shiohara et al. 2004
CdTe	Rat pheochromocytoma cells, murine, microglial cells	1 × 10^5^ cells/cm^2^	0.01 to 100 μg/mL	2 to 24 h	10 μg/mL cytotoxic	Lovric et al. 2005
CdSe-MAA, TOPO QDs	Primary rat hepatocytes		62.5-1,000 μg/mL	1 to 8 h	Cytotoxic: 62.5 μg/mL cytotoxic under oxidative/photolytic conditions	Derfus 2004
					No toxicity on addition of ZnS cap	
QD micelles: CdSe/ZnS QDs in (PEG-PE) and phosphatydilcholine	*Xenopus* blastomeres	5 × 10^9^ QDs/cell(approximately 0.23 pmol/cell)	1.5 to 3 nL of 2.3-μM QDs injected, approximately 2.1 × 10^9^ to 4.2 × 10^9^ injected QDs/cell	Days	5 × 10^9^ QDs/cell: cell abnormalities, altered viability and motility	Dubertret et al. 2002
					No toxicity at 2 × 10^9^ QDs/cell	
CdSe/ZnS amp-QDs and mPEG QDs [158]	Mice	200-μL tail vein injection	Injections, approximately 180-nM QD, approximately20-pmol QD/g animal weight	15-min cell incubations, 1 to 133 days *in vivo*	No signs of localized necrosis at the sites of deposition	Ballou et al. 2004
CdSe/ZnS-DHLA	*Dictyostelium discoideum* and HeLa cells		400 to 600 nM	45 to 60 min	No effects on cell growth	Jaiswal et al. 2003
Avidin-conjugated CdSe/ZnS QDs	HeLa cells		0.5 to 1.0 μM	15 min	No effect on cell growth and development	Jaiswal et al. 2003
CdSe/ZnS-amphiphilic micelle	Mice	Tail vein injection	60-μM QD/g animal weight, 1-μM and 20-nM final QD concentration	Not given	Mice showed no noticeable ill effects after imaging	Larson et al. 2003
CdSe/ZnS-DHLA QDs	Mice, B16F10 cells	5 × 10^4^ B16F10 cells with 10-μL QDs (approximately 10 pmol), tail vein (iv) injection	100 μL of B16F10 cells used for tail vein injection, approximately 2 × 10^5^ to 4 × 10^5^ cells injected	4- to 6-h cell incubation, mice sacrificed at 1 to 6 h	No toxicity observed in cells or mice	Voura et al. 2004
CdSe/ZnS-MUA QDs; QD-SSA complexes [162]	Vero cells	0.4 mg/mL	0.24 mg/mL	2 h	0.4-mg/mL MUA/SSA-QD complexes did not affect viability of Vero cells	Hanaki et al. 2003
CdSe/ZnS	HeLa cells	1 × 10^6^ cells	10-pmol QDs/1 × 10^5^ cells (approximately 10 nM)	10 days (cell culture)	10-nM QD had minimal impact on cell survival	Chen and Gerion 2004
CdTe aqQDs	HEK293 cells	1 × 10^5^ cells	300 or 600 nM	3 days	Nearly completely inhibited cell growth even from the very beginning	Nan Chen et al. 2012
CdTe-gelatinized/nongelatinized	PC12 cells	1 × 10^5^ cells/cm^2^	1 to 100 nM	72 h	At 1 nM,did not initiate any detrimental effects; at 100 nM, resulted in the death of all cells	Babu R Prasad et al. 2010
CdTe, CdTe/CdS, CdTe/CdS/ZnS	K562 and HEK293T human cell lines	1 × 10^5^ cells	0.2 to 3.0 μM	0 to 48 h	Cells treated with CdTe and CdTe/CdS QDs were mostly nonviable by 48 h (for all concentrations tested).	Su et al. 2009
CdSe/ZnS-PEG (EviTag T1 490 QD)	Caco-2 (human colon carcinoma) cell line	10^6^ cells/ml, 0.2 ml/well	0.84 to 105 μM	0 to 24 h	Commercially available QD demonstrated low cytotoxicity but induced cell detachment.	Wang et al. 2008
CdSe	Primary rat hippocampal neuron cells in culture	10^4^to 10^5^ cells/ml	1, 10, and 20nM	24 h	1-nM QD for 24 h showed no decrease in cell viability; in contrast, cells treated with 10- and 20-nM QD for 24 h showed decreases in cell viability on the order of 20 and 30%.	Tang et al., 2008

## Conclusions

In this review, we summarize few experiments that illustrate the high potential of QDs used for/as:

 1. labeling biomolecules and cells;

 2. tracer to follow the intracellular/extracellular dynamic of a single biomolecule/cell;

 3. localization of biomolecules *in vitro*/*in vivo*;

 4. imaging of biomolecules or cells *in vitro*/*in vivo*;

 5. assessing cell growth in damaged tissue;

 6. pH probes for the study of enzyme reaction kinetics;

 7. biomarker detection in various cancers;

 8. imaging and sensing of infectious diseases; and

 9. protein micro- and nanoarrays to the detection of cancer biomarkers.

These studies have been generated using QDs because of their small size, brightness, independence of emission on the excitation wavelength, and stability under relatively harsh environments which would be advantageous. In contrast, there are different opinions about the toxicity and fate of QDs *in vivo*. Therefore, more experiments should be done, and much more data should be available, to be sure to do clinical trials on humans.

### Future prospects

In the future, QDs will be used for identifying various categories of cancer cells, the molecular mechanisms of disease, and new drug action mechanisms, applying them in the intracellular/extracellular studies, and making new methods for biochemical assaying.

## Competing interests

The authors declare that they have no competing interests.

## Authors' contributions

SD conceived of the study and participated in its design and coordination. AA participated in the sequence alignment and drafted the manuscript. All authors read and approved the final manuscript.
